# New-Onset Atrial Fibrillation Potentially Associated With Tirzepatide: A Case Report

**DOI:** 10.7759/cureus.99651

**Published:** 2025-12-19

**Authors:** Akhtar Purvez, Mohd Mirza, Mudhasir Bashir

**Affiliations:** 1 Clinical Research, Momentum Medical Research, Charlottesville, USA; 2 Clinical Sciences, DeBusk College of Osteopathic Medicine, Lincoln Memorial University, Harrogate, USA; 3 Interventional Cardiology, Virginia Tech Carilion School of Medicine, Roanoke, USA; 4 Psychiatry and Behavioral Sciences, University of Virginia, Charlottesville, USA

**Keywords:** arrhythmia, atrial fibrillation, gip, glp-1 receptor agonist, pharmacovigilance, tirzepatide

## Abstract

Tirzepatide, a dual glucose-dependent insulinotropic polypeptide (GIP) and glucagon-like peptide-1 (GLP-1) receptor agonist, is increasingly prescribed for the treatment of type 2 diabetes and obesity. Although it is generally considered cardiometabolically beneficial, rare cardiac rhythm disturbances may occur. We describe a 62-year-old woman without a prior history of cardiovascular disease who developed new-onset atrial fibrillation (AF) shortly after initiating tirzepatide. She presented with palpitations, hypotension, and rapid ventricular response requiring intensive care, rate control, anticoagulation, and electrical cardioversion. A thorough examination showed that there was no structural heart disease, electrolyte imbalance, or endocrine disorder. Following discontinuation of tirzepatide, AF did not recur during follow-up. This case highlights the importance of clinical vigilance for arrhythmias when initiating novel incretin-based therapies.

## Introduction

Tirzepatide is a novel dual glucose-dependent insulinotropic polypeptide (GIP) and glucagon-like peptide-1 (GLP-1) receptor agonist approved for the treatment of type 2 diabetes mellitus and chronic weight management. Large randomized trials, including the SURPASS program, have demonstrated substantial reductions in glycated hemoglobin (HbA1c) and body weight compared with established therapies [1-4]. Improvements in blood pressure, inflammatory markers, and lipid profiles accompany these effects, indicating a broad cardiometabolic benefit [5-7]. Meta-analyses and real-world data further support a favorable cardiovascular safety profile, with low rates of major adverse cardiovascular events [6-9].

GLP-1 receptor agonists are known to cause small increases in resting heart rate. These changes are thought to be caused by changes in the autonomic nervous system and direct effects on the sinoatrial node [10]. Incretin-based therapies also exert various hemodynamic, metabolic, and neurohormonal effects that may influence cardiovascular physiology [11,12]. Atrial fibrillation (AF) results from intricate interactions involving atrial structural remodeling, autonomic dysregulation, and metabolic disturbance [13]. Weight reduction and improved metabolic control can reduce AF burden and recurrence, yet drug-induced arrhythmogenesis remains a recognized clinical phenomenon [14,15]. Current AF management guidelines emphasize individualized risk stratification, anticoagulation, and tailored rate or rhythm control strategies [16].

## Case presentation

A 62-year-old woman presented to our outpatient pain clinic for follow-up of chronic musculoskeletal pain. Her past medical history was significant for osteoarthritis and insomnia. She had no known history of AF, coronary artery disease, heart failure, valvular heart disease, hypertension, diabetes mellitus, thyroid dysfunction, or obstructive sleep apnea. She was a non-smoker and denied excessive alcohol or caffeine use. There was no family history of premature cardiovascular disease or arrhythmia. Her long-term medications included non-opioid analgesics and a sedative-hypnotic for insomnia. Non-opioid analgesics included the rare use of acetaminophen 500 to 1000 mg two to three times a month, as needed for pain. She was not taking steroids, opioids, nonsteroidal anti-inflammatory drugs (NSAIDs), or any other non-narcotic analgesics. She was also not on any antiarrhythmic agents, sympathomimetics, or other drugs known to provoke atrial or ventricular arrhythmias. Another provider had initiated tirzepatide therapy for weight reduction three weeks prior to this visit. She received three once-weekly subcutaneous injections at the recommended starting dose with a planned gradual uptitration. After each dose, she experienced mild nausea but no vomiting or diarrhea, and maintained adequate oral intake. Approximately 24 hours before the index clinic visit, she developed her symptoms. She had received the last dose of tirzepatide 48 hours before. These symptoms included sudden-onset palpitations associated with shortness of breath, lightheadedness, and a sensation of a "racing" heartbeat. She initially attributed these symptoms to anxiety and deferred seeking emergency care. At her scheduled clinic appointment, her vital signs showed that her blood pressure was 90/60 mmHg, her heart rate was 130-160 beats per minute, her respiratory rate was 20 breaths per minute, and her oxygen saturation was 98% on room air. She appeared anxious but was alert, oriented, and able to converse. Cardiovascular examination demonstrated an irregular rhythm without appreciable murmurs, gallops, or rubs. Jugular venous pressure was not elevated, and there was no peripheral edema. Lung auscultation revealed clear breath sounds bilaterally. Abdominal and neurologic examinations were unremarkable.

**Figure 1:**
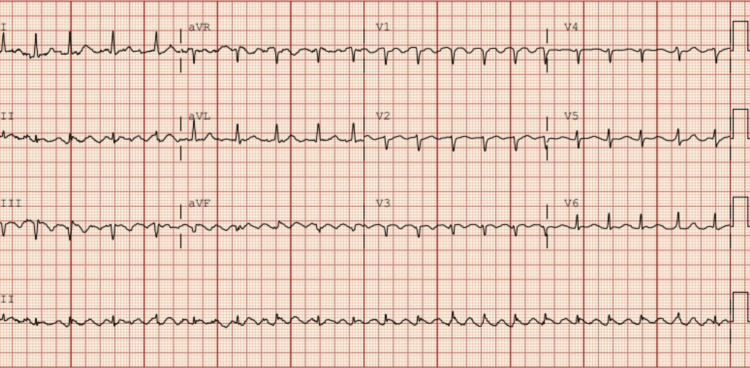
ECG showing atrial fibrillation with rapid ventricular response ECG, electrocardiogram

Given the degree of tachycardia and hypotension, she was transferred emergently to the emergency department. A 12-lead electrocardiogram (ECG) showed AF with a rapid ventricular response, a ventricular rate of approximately 130-150 beats per minute, and narrow QRS complexes without acute ischemic changes (Figure 1).

She was subsequently admitted to the intensive care unit for continuous monitoring and further management. The laboratory evaluation revealed a normal complete blood count, a normal basic metabolic panel, and normal liver function tests. Serum potassium and magnesium levels were within normal limits. The thyroid-stimulating hormone level was normal, and the high-sensitivity troponin level was not elevated. Initial screening showed no signs of infection, and a chest X-ray showed clear lung fields with no signs of cardiomegaly or pulmonary edema.

**Figure 2:**
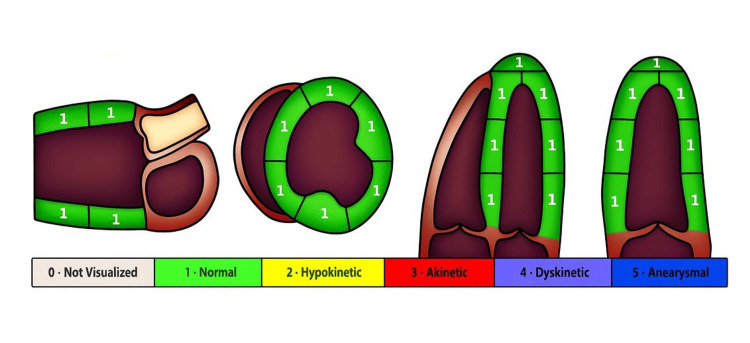
Graphic representation from the patient's transthoracic echocardiogram report

The patient underwent transthoracic echocardiography. A thorough report contained a graphic highlighting normal function (Figure 1), relevant images (Figure 2), and a text report. It revealed normal left ventricular size and systolic function, normal right ventricular function, normal sizes of both left and right atria, and no clinically significant valvular abnormalities or pericardial effusion. There was no evidence of structural heart disease that could explain the arrhythmia. Figure 2 shows the images from the transthoracic echocardiogram.

**Figure 3:**
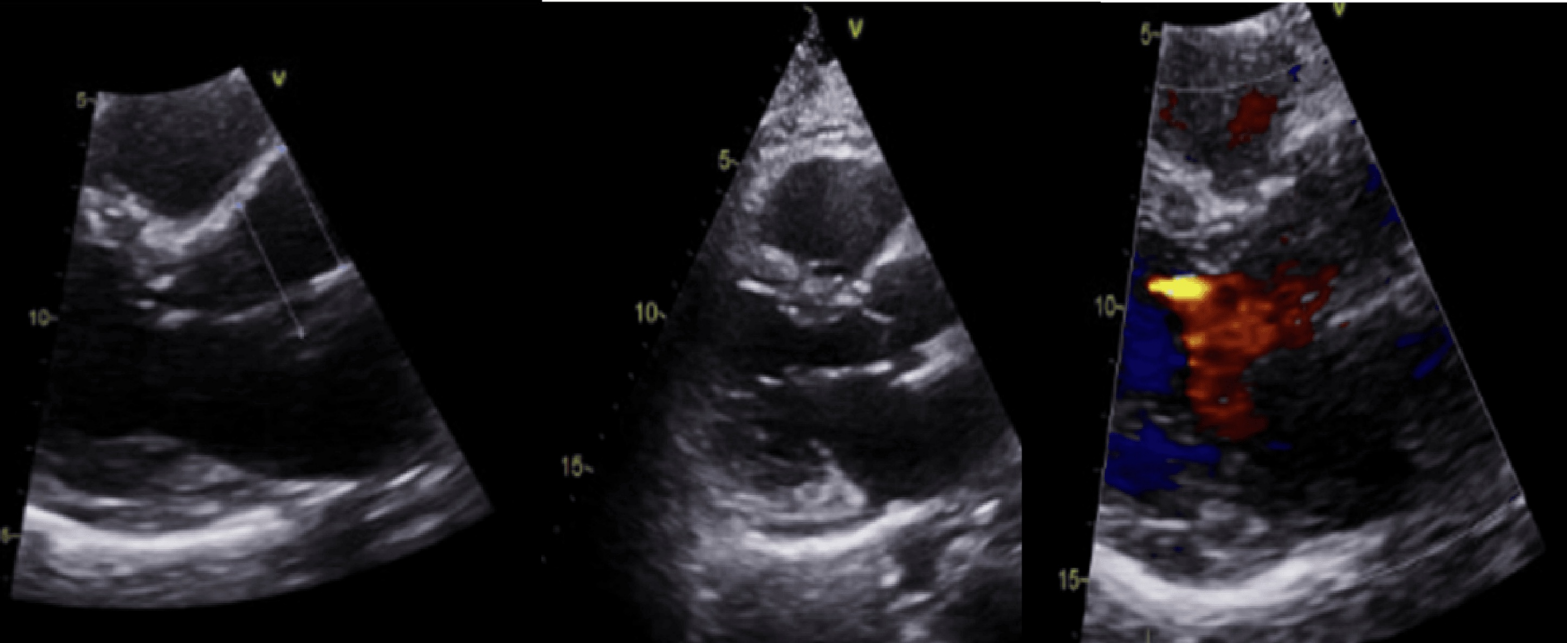
Transthoracic echocardiogram images Normal left ventricular size and systolic function, normal right ventricular function, normal sizes of both left and right atria, and no clinically significant valvular abnormalities or pericardial effusion.

Figure 3 shows the images from the transthoracic echocardiogram.

Initial rate control was achieved through intravenous therapy, which was followed by a transition to oral nadolol. Her CHA₂DS₂-VASc score was calculated as two, and she was started on apixaban for stroke prevention in accordance with guideline-directed management [16]. A transesophageal echocardiogram performed prior to cardioversion revealed no left atrial appendage thrombus. Synchronized direct-current cardioversion successfully restored the patient's sinus rhythm on the first attempt.

Tirzepatide was discontinued at the time of hospitalization due to concerns about a potential drug-related contribution to the arrhythmia. The patient maintained sinus rhythm for the rest of her hospital stay and was sent home on nadolol and apixaban, with a plan for close follow-up. After several months of outpatient follow-up, the patient reported a resolution of palpitations and no recurrent episodes of AF. Serial ECGs confirmed a persistent sinus rhythm. The patient's clinical course and the diagnostic ECG obtained at presentation are summarized in Figure 1 [17-20].

## Discussion

This case describes new-onset AF in close temporal association with the initiation of tirzepatide in a woman without structural heart disease or traditional AF risk factors. In large randomized trials, tirzepatide has been shown to improve glycemic control and facilitate weight loss. It also has beneficial effects on blood pressure and lipid profiles [1-4,5-7]. Meta-analyses and real-world evidence support a generally favorable cardiovascular safety profile and a low incidence of major adverse cardiovascular events [6-9]. Table 1 lists the incidence of adverse events associated with the GLP-1 class of medications.

**Table 1:** Common adverse events reported with GLP-1 receptor agonists

Adverse event	Incidence range (%)	Frequency category	Notes
Nausea	20-40% [12,13]	Very common	Most frequent; dose-dependent, early-onset
Diarrhea	10-20% [12,14]	Very common	Improves with continued therapy
Decreased appetite	10-20% [12]	Very common	Contributes to weight loss
Vomiting	5-15% 12,14]	Common	More frequent with higher doses
Constipation	5-10% [13]	Common	Typically mild and self-limited
Dyspepsia/upper abdominal discomfort	5-10% [12,15]	Common	Often overlaps with nausea or bloating
Abdominal pain	3-10% [13]	Common	Usually non-specific and transient
Injection-site reactions	5-10% [12]	Common	More frequent with shorter-acting agents
Headache	5-10% [13,16]	Common	Rarely leads to discontinuation
Dizziness	3-5% [14]	Common	Often related to reduced oral intake
Gallbladder-related events	1-3% [13,15]	Common-uncommon	Higher risk with rapid weight loss
Tachycardia/palpitations	<5% [12,17]	Common-uncommon	Reflects class-related heart rate increase
Acute pancreatitis	<1% [18]	Uncommon/rare	Rare; causal link uncertain

Nonetheless, GLP-1 receptor agonists, including those with dual GIP activity, consistently produce modest increases in resting heart rate, which may reflect autonomic activation and effects on the sinoatrial node [10-12]. AF results from a multifaceted interaction of atrial structural remodeling, fibrosis, inflammation, autonomic dysregulation, and metabolic stress [13]. Weight loss and better management of cardiometabolic risk factors can lower the burden of AF and improve rhythm-control outcomes. However, medications can also cause AF in people who are already at risk [14-16]. Observational studies and case reports have raised concern that some glucose-lowering agents may influence arrhythmia risk. However, data for incretin-based therapies essentially suggest neutrality or benefit at the population level [11,12,17-20]. Drug-induced AF is a well-recognized clinical entity. Kaakeh and colleagues provide a comprehensive review of medications associated with AF, outlining mechanisms such as triggered activity, reentry, ion-channel modulation, and autonomic imbalance [21]. Several of these mechanisms overlap conceptually with the known physiologic actions of incretin-based therapies, supporting the plausibility that tirzepatide could act as a trigger in a susceptible substrate. Environmental and dietary factors may also precipitate AF. Case reports describe AF episodes following ingestion of monosodium glutamate (MSG) and aspartame, suggesting a potential contribution of excitatory neurotransmitter pathways and autonomic perturbation [22]. Public health discussions have driven home the need for uniform MSG labeling regulations to support consumer protection, particularly for individuals who may be sensitive to such exposures [23]. In a previously reported case from our group, AF occurred in temporal association with MSG consumption in a 78-year-old woman, further illustrating how exogenous dietary triggers can precipitate AF in predisposed patients [24]. In the present case, common precipitating factors for AF, including structural heart disease, electrolyte abnormalities, hyperthyroidism, acute coronary syndrome, and infection, were systematically excluded. The patient had no prior history of AF, and transthoracic echocardiography demonstrated normal atrial size and ventricular function. AF emerged after several weeks of tirzepatide therapy and has not reappeared following the cessation of the medication and the reestablishment of sinus rhythm. Taken together, these observations raise suspicion for a possible tirzepatide-related arrhythmic event. The Naranjo Adverse Drug Reaction Probability Scale yielded a score in the "possible" range for this case, suggesting a conceivable but not conclusively confirmed causal relationship (Table 2). Clinicians must recognize the potential for arrhythmic symptoms in patients commencing tirzepatide, especially in individuals with preexisting autonomic sensitivity or subclinical atrial pathology. Guideline-directed therapy, including appropriate anticoagulation and individualized rate- or rhythm-control strategies, remains the cornerstone of AF management [16].

**Table 2:** Naranjo adverse drug reaction probability scale Source: [25]

Question	Response	Score
Did the event appear after the drug was given?	Yes	+2
Are alternative causes present?	Possible but not identified	+1
Did symptoms improve after stopping?	Yes, no recurrence	+1
Did the reaction recur with re-exposure?	Not attempted	0
Are previous reports available?	Yes, arrhythmias reported	+1

Given the expanding use of tirzepatide and other incretin-based agents in both diabetic and non-diabetic populations, further pharmacovigilance, registry studies, and mechanistic research are warranted to clarify their electrophysiologic safety profile and identify patient subgroups at increased risk.

## Conclusions

This case highlights a possible association between tirzepatide and new-onset AF in a woman without structural heart disease or traditional AF risk factors. Although causality cannot be confirmed, the temporal relationship, exclusion of common secondary causes, and absence of recurrence after discontinuation of tirzepatide raise concern for a drug-related effect. Clinicians should maintain a high index of suspicion for arrhythmias in patients who report palpitations or related symptoms after starting tirzepatide or other incretin-based therapies. Careful monitoring, patient education, and prompt evaluation of new cardiac symptoms are recommended. Continued pharmacovigilance and future research are needed to better define the arrhythmogenic potential of dual incretin agonists and to guide safe prescribing in at-risk populations.
